# Editorial: Advancements in understanding nasal airflow and improved success of intranasal surgery

**DOI:** 10.3389/fsurg.2026.1963390

**Published:** 2026-09-29

**Authors:** Johan Hellgren, Sverre Steinsvåg

**Affiliations:** 1 Department of Otorhinolaryngology, Head & Neck Surgery, Institute of Clinical Sciences, University of Gothenburg, Gothenburg, Sweden; 2 Department of Otorhinolaryngology, Sjukhusen i Väster, Region Västra Götaland, Gothenburg, Sweden; 3 Departments Of Otolaryngology, Head and Neck Surgery, Sørlandet Hospital and Haukeland University Hospital, University of Bergen, Bergen, Norway

**Keywords:** advancements, Editorial, intranasal surgery, nasal airflow, rhinologic surgery, Turbinate surgery

The nose is an important organ in the human body, and diseases and anatomical anomalies may have substantial impact on patient's daily function and physical and mental well-being. Even though, readily accessible for examinations and studies, surgical treatment of the nose and the nasal passages are still a significant challenge (1). Understanding the diverse and dynamic surgical anatomy as well as the function of the upper airway and how surgical interventions can change function are key issues. In this special Research Topic, “Advancements in Understanding Nasal Airflow and Improved Success of Intranasal Surgery” important new data and insights are presented in three clinical studies and two review papers.

The nasal-to-oral airflow pressure ratio was studied with a novel device to improve correct measures of nasal obstruction (Wang et al.). Nasal obstruction is a subjective sensation and not always represented by a corresponding increased nasal airway resistance. In addition, the correlation between patient rated outcome measures (PROMS) and measurements of nasal airflow and resistance are not consistent between studies (2–4). In the assessment of patients for surgery as well as in cases of treatment failure, correct measures of nasal airflow and resistance can contribute valuable information. In follow-up studies after septoplasty the satisfaction rate measured with different PROMS is 2/3-3/4 of patients after up to 12 months post-surgery (5, 6). This shows that both in the selection of patients for surgery and in the follow up, exact tools for nasal airflow measurements are needed.

Most studies of nasal function are conducted on patients with sino-nasal diseases compared to healthy controls. In elite enduro athletes it is possible to study nasal function under extreme exercise conditions and its contribution to the metabolic demand. In the present study, repeated measures of nasal airflow and resistance were performed during increased exercise load to a maximum (Bergqvist et al.) New insights are presented on the contribution of nasal breathing to extreme physical performance.

Nasal obstruction as well as upper airway inflammatory diseases are linked to sleep disordered breathing (7). It has proven difficult to identify the exact mechanism by which nasal obstruction is a driver in the upper airway collapse during sleep, but it has been shown that nasal surgery can improve the quality of sleep and subjective symptoms of sleepiness (8). In a recent clinical study, the effect of septoplasty and turbinate reduction on subjective sleep parameters was studied. Nasal surgery has been proven to facilitate CPAP use and to lower the effective CPAP pressure but not to cure obstructive sleep apnea. (Alves de Sousa et al.)

The nasal valve is the most critical region for nasal airflow. More than 50% of the total airway resistance is in the most anterior 2 cm of the nose (9). The nasal valve can be congenitally narrow, the side walls of the vestibulum can be weak and collapse during inspiration, and it may be narrow after surgery or trauma. In line with Poiseuille's law for laminar flow, even minor narrowing of the nasal valve may have major negative impact on nasal airflow. Options for treatment include reduction of mucosal edema by saline irrigation or topical nasal steroids, external or internal nasal dilators or surgical reconstruction of the valve area, in some cases combined with surgery on the nasal septum and the turbinates (Pirola et al.).

The external nose is important for our identity from an esthetic standpoint, and surgery of the external nose is a common surgical procedure. The external nose is also important in defining the outlines of the upper airway and rhinoplasty is therefore often considered in surgery relieving nasal obstruction when septoplasty and turbinoplasty alone are not adequate. It is therefore crucial to assess the potential impact of rhinoplasty for esthetic reasons on the upper airway so that alterations of the external nose do not impair the nasal airway. In an interesting review the authors review the evidence comparing preservation rhinoplasty versus conventional (Barnawi et al.).

As Editors, we believe this research topic will offer new and interesting reading for all clinicians and researchers with an interest in the nose.

Sverre Steinsvåg Johan Hellgren.
